# A spatiotemporal XGBoost model for PM_2.5_ concentration prediction and its application in Shanghai

**DOI:** 10.1016/j.heliyon.2023.e22569

**Published:** 2023-11-23

**Authors:** Zidong Wang, Xianhua Wu, You Wu

**Affiliations:** School of Economics and Management, Shanghai Maritime University, Shanghai 201306, China

**Keywords:** Extreme gradient boosting model (XGBoost), PM2.5, Signal decomposition, Kriging interpolation

## Abstract

This paper innovatively constructed an analytical and forecasting framework to predict PM_2.5_ concentration levels for 16 municipal districts in Shanghai. By means of XGBoost parameters adjustment, empirical mode decomposition, and model fusion, improvements are made on XGBoost prediction accuracy and stability so that prediction deviation at extreme points can be avoided. The main findings of this paper can be summarized as follows: 1) Compared with the original model, the goodness of fit of the modified XGBoost model on the test set increased by 17 %, and the root mean square error decreased by 28 %; 2) The variation of PM_2.5_ concentration in Shanghai has a significant seasonal (cyclical) effect, and its fluctuation period is 3 months (a quarter). In winter, the frequency of extreme value points is significantly higher than that in other seasons; 3) In terms of spatial distribution, the PM_2.5_ concentration in the central city of Shanghai is higher than that in the rural areas, and the PM_2.5_ concentration gradually decreases from center city to the surrounding areas. The innovation and contribution of this paper can be summarized as follows: 1) EEMD algorithm verified by SSA was used to decompose the original model without reconstructing all subsequences and get the best weighing among each part of the hybrid model by using variable weight assignment; 2) The city was cut into pieces according to administrative districts in avoid of the duplicate analysis when utilizing advised Kriging interpolation; 3) IDW method was applied to verified Kriging interpolation to increase the accuracy; 4) The latitude and longitude were innovatively converted into the arc length of the corresponding spherical surface; 5) Hierarchical analysis method was used to obtain the order of importance among the PM_2.5_ monitoring stations, which could improve the accuracy and achieve dimension reduction.

As a Principal Investigator, He has 7 national research projects such as “The Major Research Plan of National Social Science Foundation (18ZDA052): Research on the Control Optimization of Air Pollutant Emission in the Era of Big Data”, “Training Program of the Major Research Plan of the National Science Foundation of China (91546117): Research on Integration Method of Big Data of Meteorological Disasters for Supporting Emergency Decision-making” and etc. He is also a member of Steering Committee of Economics Education (2018–2022), MOE, China.

## 1 Introduction

The fluctuation of PM_2.5_ is affected by many factors. There are more and more researches on PM_2.5_ prediction using machine learning methods, especially the extreme gradient boosting model (XGBoost). Random forest model [1], enhanced long short-term memory neural network model [2] and LightGBM model [3] performed effective in prediction of short-term PM_2.5_ concentration changes, which indicates an advantage of using machine learning algorithms to predict short-term changes in PM_2.5_, especially those data sets with high spatial and temporal resolution.

The methods used in current research are mainly divided into two kinds: conventional statistical methods, and machine learning methods. Early research on prediction models mainly focused on methods related to regression analysis, like data-driven ordinary differential equation (ODE) model established by Wang et al. [4] and an improved grey correlation analysis method called grey multivariate convolution model (GMCN(1,*N*)) applied by Zhang et al. [5]. What's more, the relationship between PM_2.5_ and other pollutants came to the fore [6], while sequence information be extracted to improve the precision by using complementary integrated empirical mode decomposition method --- after reconstructing, the predicted values of the decomposed subsequences could perform better in predicting time sequence like PM_2.5_ concentration [7]. Parameter optimization method like principal component analysis (PCA) used by Sun and Sun [8] was gradually added to prediction model like long-short-term memory (LSTM) used by Xu and Yoneda [9], in order to compose hybrid model, which could extremely improve the predicting capability.

As a relatively advanced gradient boosting algorithm, XGBoost has attracted much attention in recent years. With the development of machine learning technology, scholars began to apply XGBoost to predict PM_2.5_ concentration. Early studies mainly used XGBoost alone for empirical analysis, and compared it with other algorithms to verify its advantages [10]. In short-term prediction, especially when we get the data set of hourly PM_2.5_ mass concentration [11], XGBoost has a smaller percentage error than the LSTM network [12], which may be ascribed to the difference in training mechanism between them. When taking more stations into consideration [13], the prediction results of constructed XGBoost network always show strong robustness and high accuracy. Combined other predicting model and XGBoost to establish a hybrid model for forecasting of PM_2.5_ could largely improve the accuracy in some extreme point---in these days the PM_2.5_ concentration is largely higher or lower than the day before or later [14]. The establishment of a reliable PM_2.5_ prediction system based on distributed interactive platform is conducive to real-time prediction [15].

Optimization of the XGBoost prediction data itself can improve the prediction performance of the model. There are two methods for scholar to do the research when preprocessing PM_2.5_ sequence before predicting. How to fill in these missing values by improving XGBoost is the focus of scholars. The handling of missing values is a common method in predicting PM_2.5_ concentration, especially the problem of non-random missing data [16,17]. For example, Wang et al. [18] used wavelet decomposition (WD) to solve the Aerosol Optical Depth (AOD) missing problem in PM_2.5_ prediction.

Another method to improve the prediction performance of the model is analyze the PM_2.5_ sequence before predicting. This study uses empirical modal decomposition (EMD) to do the preprocessing [19]. EMD for PM_2.5_ sequences preprocessing can improve the prediction accuracy of machine learning models. Furthermore, with the EMD method for PM_2.5_ prediction, Jin et al. [20] reconstructed the decomposed sequences by frequency, then predicted them separately, and finally merged the frequency sequences to obtain the predicted values. By doing so, the root mean square error on the test set is significant reduced. What's more, the prediction efficacy of neural network models could also be enhanced by EMD model [21]. Thus, we apply EMD method to improve the predicting accuracy of our model.

The spatial distribution of PM_2.5_ has long been a matter of interest to scholars. The model prediction of PM_2.5_ distribution can help to discover its spatial characteristics. Liu et al. [22] found that the spatial distribution of PM_2.5_ was significantly different between urban and rural areas. The division of the study area according to administrative areas is a common practice of regional partitioning [23] when analyzing the spatial-temporal distribution of PM_2.5_. What should also be noted in partitioning the study area is the altitude differences among each sub-area (e.g. plain area, river delta, the bottom of basin etc.) will also affect the distribution of PM_2.5_ concentration [24]. Thus, we innovatively utilize the inverse distance weighting (IDW) method to deal with data sets and we use kriging interpolation method to show the difference of PM_2.5_ concentration in 16 districts in Shanghai. The combination of these two methods performs well.

The current research on XGBoost mainly focuses on how to build a combined model to improve XGBoost. One way is to combine multiple methods. For example, ridge regression [25] and three-dimensional landscape pattern index (TDLPI) [26] both being added to XGBoost could make up for the low data monitoring accuracy of the air quality detector. Multi-source data including spatiotemporal, periodic, meteorological, vegetation, human and topological features could be integrated into the hybrid predicting model [[27], [28], [29]], which could significantly contribute to the prediction accuracy if all variables were independent with each other. Another way is to adjust the XGBoost model parameters by using grid search method or genetic algorithm to optimize the predictions [30]. In addition to its good performance in the prediction of continuous variables, XGBoost is also widely used in image recognition and classification [31,32]. However, up to date, the literature concerning searching the best weighing between each part of the combined model is still lacking. One innovation of us is that we efficiently find the best parameter of this gradient boosting algorithm and get the best weighing.

In general, machine learning algorithms tend to replace traditional statistical methods in the prediction of PM_2.5_. Among them, gradient boosting algorithms are increasingly used for PM_2.5_ prediction. In terms of variables affecting PM_2.5_, the relationship between AOD data and PM_2.5_ has become a research hotspot, but there is little discussion on other variables except meteorological factors and other pollutants. Thus, one of the contributions is that we put air pollutant indicators, meteorological environmental factors and macroeconomic indicators into XGBoost model in order to fully acquire the relationship between PM_2.5_ and all these possible variables. Numerous studies have shown that fusion models can improve the accuracy of predictions. Therefore, the focus of this paper is to select a suitable model to fuse with XGBoost to improve the prediction effect. What's more, we find that the fusion of gradient boosting algorithms LightGBM, GBDT and XGBoost can enhance the complementarity of the first-layer prediction model to a certain extent. Furthermore, we weighted the predicted PM_2.5_ values by the three gradient lifting algorithms, and found that the new sequence formed was closer to the actual PM_2.5_ value.

## 2 Research methods and modeling

### 2.1 Important parameter choice of main model

#### 2.1.1 Revised empirical mode decomposition

PM_2.5_ concentration data are nonlinear non-stationary time series with daily fluctuations. When there are too many subsequences decomposed by EMD, or modal aliasing occurs after decomposition, the SSA method can be applied to decompose the sequence. After the window width is specified, the decomposed submatrix is reconstructed according to Eq. (1):

(1) $$e_{K}=\left\{ \begin{array}{cc} \frac{1}{K}\cdot \sum_{P=1}^{K}e_{P,K-P+1}^{*}, & 1\leq K<m\\ \frac{1}{m}\cdot \sum_{P=1}^{m}e_{P,K-P+1}^{*}, & m\leq K<n\\ \frac{1}{N-k+1}\cdot \sum_{P=K-n+1}^{N-n+1}e_{P,K-P+1}^{*}, & n\leq K<N \end{array}\right.$$

where, *e*_*P*_ is the decomposed subsequence; *e*_*K*_ is the reconstructed high-frequency, low-frequency and residual sequences; *m* and *n* are the given widths. The sequences with low correlation (correlation coefficients between all the decomposed subsequences and the original PM_2.5_ sequence less than 0.15) will be removed. According to the index value, the subsequences are classified into three categories: high frequency, low frequency and residual. After that, the three reconstructed sequences are predicted respectively.

#### 2.1.2 The parameter choice of XGBoost modeling

Given that XGBoost uses a second-order Taylor expansion, a quadratic function can improve the accuracy of the approximation. At this time, the default regular term selected is L_2_, that is, the square of the norm of the second fundamental form. Chen and Guestrin [33] once gave the regular term of the gradient boosting algorithm.

(2) $$\Omega \left(f\left(t\right)\right)=\gamma \cdot T+\frac{1}{2}\cdot ƛ\cdot {\left\|w\right\|}^{2}$$

where Ω(f(t)) is the complexity of the model in the *t*-th round iteration, *w* is the weight of the leaf node, γ is the learning rate, *T* is the number of the leaf nodes, and ƛ is the L_2_ regularization term for the weight.

At the same time, in Eq. (2), the L_1_ regularization method makes it easier for the parameter ƛ to be 0, so it is not as effective as L_2_ for non-sparse dependent variables. In addition, since the conditions for L_1_ to obtain the extreme value at point 0 are looser than that for L_2_, the number of optimal parameters will increase, or it will fall into a local optimal solution. Thus, the XGBoost model should choose the square term as the regular term.

The innovation lies that we remove those sequences which have lower relationship with original sequence and complete incomplete reconstruction in EEMD-XGBoost model when prediction. What's more, we analyze non-sparse PM_2.5_ data set and sparse PM_2.5_ data at different air quality monitoring stations by using L2 regularization method and L1 regularization method separately, which will affect the learning rate γ in XGBoost model. What we talked above could improve the accuracy of prediction.

**Fig. 1:**
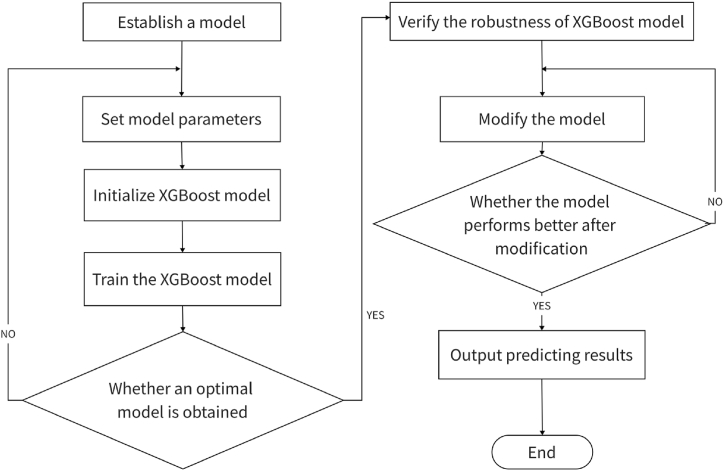
XGBoost modeling flow chart.

The specific modeling process is shown in Fig. 1.

### 2.2 Modeling steps

#### 2.2.1 Establishment of a spatiotemporal XGBoost model modified by empirical mode decomposition

**Fig. 2:**
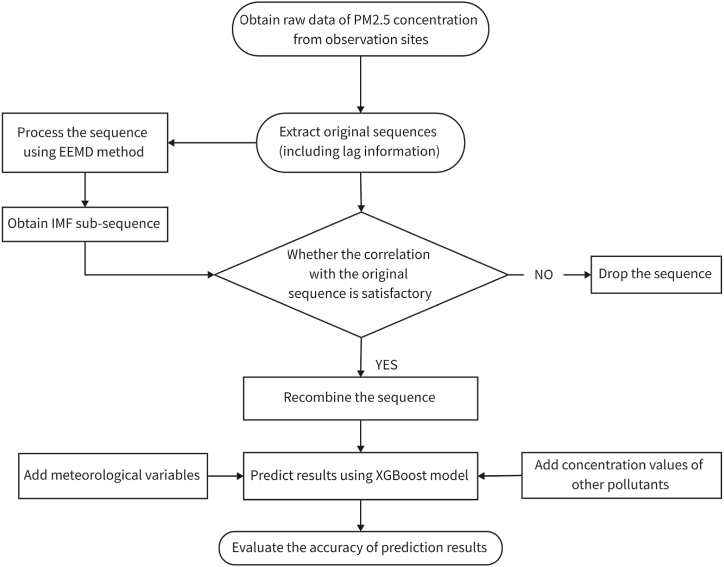
EEMD-XGBoost modeling flow chart.

First, the parameters of the model are adjusted to determine the optimal parameters of XGBoost. Next, the original PM_2.5_ concentration sequence after signal decomposition is substituted into the model as a dependent variable for prediction. The EMD-based modeling process is shown as in Fig. 2.

**Fig. 3:**
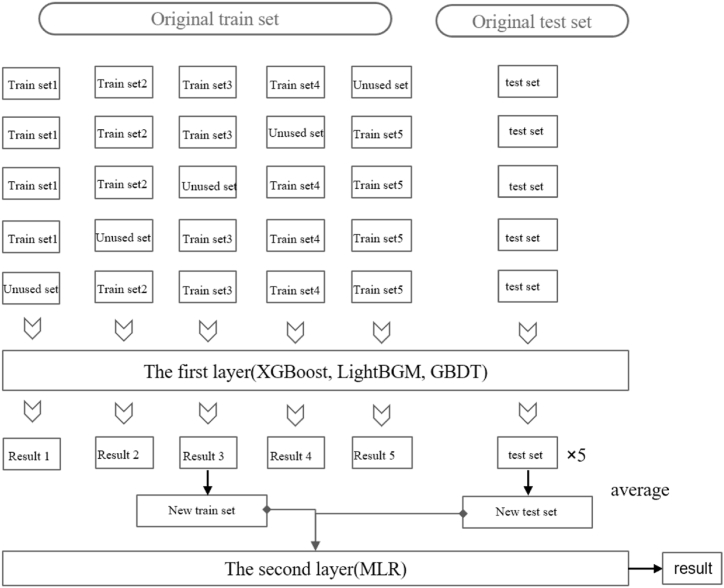
The process of dealing with data by fusion model.

In Fig. 2, EEMD is to perform empirical mode decomposition on the original sequence, and the set of sub-sequences obtained is denoted as IMF. Then, the correlation between sub-sequences and the original PM_2.5_ sequence is checked. Each subsequence is predicted and analyzed by XGBoost, and finally the predicted sequence is recombined to obtain the predicted value of PM_2.5_ sequence. Next, a fusion model of the gradient boosting algorithm is constructed to enhance the stability of the XGBoost model, as set in Fig. 3.

As shown in Fig. 3, the three gradient boosting methods are integrated, and then combined with the multiple linear regression for secondary prediction. The prediction results of the integrated model and XGBOOST are compared. Herein, 5-fold cross-validation is adopted. According to the time sequence, 20 % of the data is set aside each time, and the remaining data is used for training. The XGBoost, LightGBM, and GBDT are respectively used as the first layer of the integrated model for prediction, and the error on the training set and test set is calculated. Then the first total sequence prediction value is obtained by weighted average. The method of variable weight assignment (Eq. (6)–(8)) is adopted to determine the weight to correct the prediction error at the extreme point.

(3) $$f\left(X_{i}\right)=\frac{1}{n}\cdot \sum_{i=1}^{3}W_{i}\left(t\right)\cdot f_{i}\left(X_{i}\right)$$

where $W_{i}\left(t\right)$ is the weight of the *i*-th model at time *t*,

(4) $$W_{i}\left(t\right)=\frac{\frac{1}{\epsilon_{l}\left(t\right)}}{\frac{1}{\sum_{i=1}^{3}\overset{═}{\epsilon_{l}\left(t\right)}}}=\frac{\sum_{i=1}^{3}\overset{═}{\epsilon_{l}\left(t\right)}}{\epsilon_{l}\left(t\right)}$$

where $\epsilon_{l}\left(t\right)$ is the prediction error of the *l*-th model at time *t* and it satisfies $W_{i}\left(t\right)>0$. The prediction results of XGBoost model with variable weight modification can be obtained by substituting Eq. (4) into Eq. (3),

(5) $$s.t.\sum_{i=1}^{3}W_{i}\left(t\right)\cdot f_{i}\left(X_{i}\right)$$

In view of the fact that a weight can be obtained at each observation moment (there may be errors), the weight at each moment is adjusted by means of residual weighting. Then the corresponding optimal weight is calculated by

(6) $$\begin{array}{cc} W_{j}\left(t\right)=\frac{1}{m}\cdot \sum_{k=1}^{m}W_{j}\left(t-k\right)\text{,} & m=3 \end{array}$$

where the weight before the correction at time *t* is obtained by weighting the weight coefficients of the three adjacent periods. At the same time, the absolute error between the predicted value and the actual value at this time and the next period of this time can be calculated, i.e.,

(7) $$e_{i,t}=\sum_{i=1}^{n}W_{i}\left(t\right)\cdot f_{i}\left(X_{i}\right)-f\left(t\right)$$

(8) $$e_{i+1,t}=\sum_{i=1}^{n}W_{i+1}\left(t\right)\cdot f_{i+1}\left(X_{i+1}\right)-f\left(t\right)$$

The values of residual errors $e_{i,t}$ and $e_{j,t}$ are compared. If $e_{i,t}<e_{j,t}$, the weight combination of the previous moment with small error replaces the weight combination of the current period; if $e_{i,t}\geq e_{j,t}$, the weight of the current period is retained. The weight combination of three periods is considered, which enhances the stability of the model. In this way, EEMD-XGBoost completes fusion and adaptive weight adjustment.

#### 2.2.2 Importance ranking and spatial effect analysis

The XGBoost model can solve classification and regression problems, and explore the influence of various factors on PM_2.5_ concentration. Since PM_2.5_ concentration is a continuous variable, the dependent variable needs to be classified first. In the process of outlier processing, the observation points greater than or less than 1.5 standard deviations from the mean are regarded as outliers Therefore, the values calculated from Eqs. (9), (10) are the upper and lower thresholds for determining whether a certain point is an outlier,

(9) $$y_{L}=\bar{y}-1.5\cdot \delta_{y}$$

(10) $$y_{U}=\bar{y}+1.5\cdot \delta_{y}$$

where *y*_*L*_ and *y*_*U*_ are the upper and lower limits of the reasonable estimation interval of *y*, respectively, and $\delta_{y}$ is the standard deviation of the PM_2.5_ concentration sequence. All values in the concentration sequence are traversed to determine whether the value is an outlier. Outliers greater than *y*_*U*_ are marked as 1, the outlier smaller than *y*_*L*_ as −1, and the normal values as 0. This categorical variable is regarded as a dependent variable.

In order to analyze the interaction between measured PM_2.5_ concentration values at each observation point, the predicted PM_2.5_ concentration values are modified by inverse distance weighting. The steps to construct the distance weight matrix are as follows: the longitude and latitude coordinates of 19 monitoring stations in Shanghai are obtained by accurate positioning with the help of Google Map. The latitude and longitude can be converted into the arc length of the corresponding spherical surface through the spherical coordinate system. Let the coordinates of a point in space be *P*(*X*,*Y*,*Z*), where *X* and *Y* are the coordinates of the observation point in the space cartesian coordinate system, and *Z* is the height of the point from the horizontal ground. Let (*e*,*n*) be the latitude and longitude of a point *A* on the earth's surface and the position *P* can be obtained from Eqs. (11)–(13):

(11) x=R·cos(n)·cos(e)

(12) y=R·cos(n)·sin(e)

(13) z=R·sin(n)

**Fig. 4:**
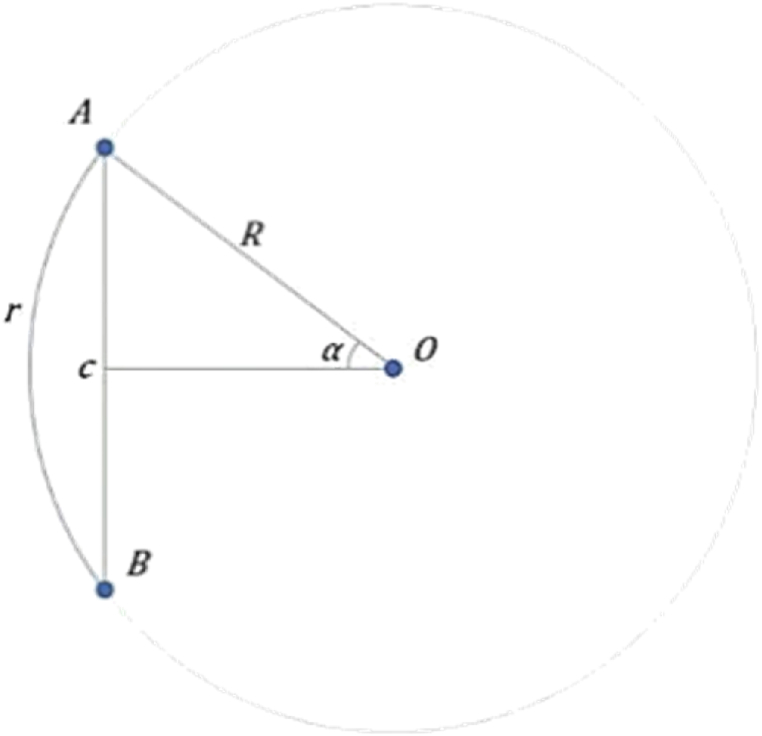
Calculation of the real distance between two points *A* and *B*.

The radius of the earth is 6371 km, denoted as *R*. The length of the minor arc corresponding to the string *AB* (the relationship of each variable is shown in Fig. 4).

Namely, the distance between stations *A* and *B*, can be obtained by Eqs. (14) – (16):

(14) c=2·R·sin(α)

(15) $$\alpha =\arcsin\;\left(\frac{c}{2\cdot R}\right)$$

(16) r=2·α·R

Wu et al. [34] utilized the inverse distance weighting (IDW) method to determine the weight of the pollution from a wildfire in the studied area contributed to overall PM_2.5_ concentration of rest of the region. Next, the inverse distance weighting method is used to reprocess the predicted values of all stations, and the process is shown in Eqs. (17), (18):

(17) $$P_{d}=\frac{\sum_{i=1}^{n}\frac{P_{i}}{d_{i}^{2}}}{\sum_{i=1}^{n}\frac{1}{d_{i}^{2}}}$$

(18) $$P_{t}=t$$

where *i* is the *i*-th station around and *t* is the observation time. The estimation accuracy of the model can be improved by introducing spatial factors. Regarding the research on distance weight, some scholars proposed the bandwidth selection method [15]:

(19) $$W_{i,j}=\left\{ \begin{array}{cc} {\left[1-{\left(\frac{d_{ij}}{b_{j}}\right)}^{2}\right]}^{2}, & d_{ij}<b_{j}\\ 0, & d_{ij}>b_{j} \end{array}\right.$$

(20) $${\text{PM}}_{2.5}\left(u_{i},v_{i}\right)=\sum_{j=1}^{n}W_{i,j}\cdot F_{j}\left(X_{i}\right)$$

where *n* is the total number of stations; $\left(u_{i},v_{i}\right)$ are the spatial coordinates; *i* and *j* are stations; $b_{j}$ is the bandwidth, kilometer; $W_{i,j}$ is the weight. The PM_2.5_ concentration at station *i* is obtained by weighting. When the distance between two stations is smaller than the given bandwidth, the station does not participate in the weighting. Wu et al. [34] applied Kriging interpolation to study the differences in PM_2.5_ concentrations, which provides a method for studying the spatial network distribution of PM_2.5_ concentrations. In our research, the revised Kriging interpolation method is used to fill in the predicted value of the whole Shanghai, and the main steps are as follows:

Let *Z*_0_* be the PM_2.5_ concentration to be estimated, and *Z*_0_ and *Z*_*i*_ be the PM_2.5_ concentrations at the known and unknown stations, respectively. First, the cost function *J* is introduced, and then the calculation formula of PM_2.5_ concentration at unknown station is substituted into *J* to obtain Eq. (22):

(21) $$Z_{0}^{*}\left(x_{0},y_{0}\right)=\sum_{i=1}^{n}\lambda_{i}\cdot Z\left(x_{i},y_{i}\right)$$

(22) $$J=Var\left(Z_{0}^{*}-Z_{0}\right)$$

(23) $$J=\sum_{i=0}^{n}\sum_{j=0}^{n}\lambda_{i}\cdot \lambda_{j}Cov\left(Z_{i},Z_{j}\right)-2\cdot \sum_{i=1}^{n}\lambda_{i}\cdot Cov\left(Z_{i},Z_{0}\right)+Var\left(Z_{0}\right)$$

Therefore, the concept of semi-variance is introduced as in Eq. (23), where $\lambda_{i}$ is the combination coefficient which needs to satisfy the constraints of Eq. (24):

(24) $$r_{ij}=\sigma^{2}-Cov\left(x_{i},y_{i}\right)$$

(25) $$\sum_{i=1}^{n}\lambda_{i}=1$$

Based on the obtained distance between any two monitoring points, Gaussian function is chosen to determine the semi-variance between any two points. The relationship between distance and semi-variance is shown in Eq. (26):

(26) $$Cov\left(Z_{i},Z_{j}\right)=f\left(\sqrt{{\left(x_{i}-x_{j}\right)}^{2}+{\left(y_{i}-y_{j}\right)}^{2}}\right)=f\left(x\right)=a\cdot \exp\left(-\frac{{\left(x-b\right)}^{2}}{2\cdot c^{2}}\right)$$

where *a*, *b*, and *c* are all constant.

Eq. (26) is used as the Lagrangian operator to derive the cost function in Eq. (27), and the equation system is obtained as below:

(27) $$\left\{ \begin{array}{c} r_{i0}-\lambda_{i}\cdot r_{ij}=0\\ \sum_{i=1}^{n}\lambda_{i}=1 \end{array}\right.$$

The combination coefficients $\lambda_{1}$, $\lambda_{2}$, …, $\lambda_{n}$ under the minimum estimated variance can be obtained by solving equation system (27). By substituting these coefficients into Eq. (21), the PM_2.5_ concentration at any location in Shanghai can be estimated. Then the PM_2.5_ predictions of all stations in Shanghai can be obtained.

The solution of Equation (30) must satisfy the two constraints (28) and (29) (Kriging interpolation conditions):

(28) $$\underset{\lambda_{i}}{\underbrace{\min}}Var\left(Q_{d}\left(x_{0}\right)-\sum_{i=1}^{n}\lambda_{i}\cdot Q\left(x_{i}\right)\right)$$

(29) $$E\left(\bar{Q_{d}\left(x_{0}\right)}-\sum_{i=1}^{n}\lambda_{i}\cdot \bar{Q\left(x_{i}\right)}\right)=0$$

where *Q*_*d*_(*x*_0_) is the spatial PM_2.5_ attribute of Shanghai, and $\lambda_{i}$ is the weighted combination coefficient of five observation stations.

After filling by Kriging interpolation, the predicted concentration of PM_2.5_ in Shanghai at each time can be obtained.

### 2.3 Data Preparation

#### 2.3.1 Introduction to the study area

**Fig. 5:**
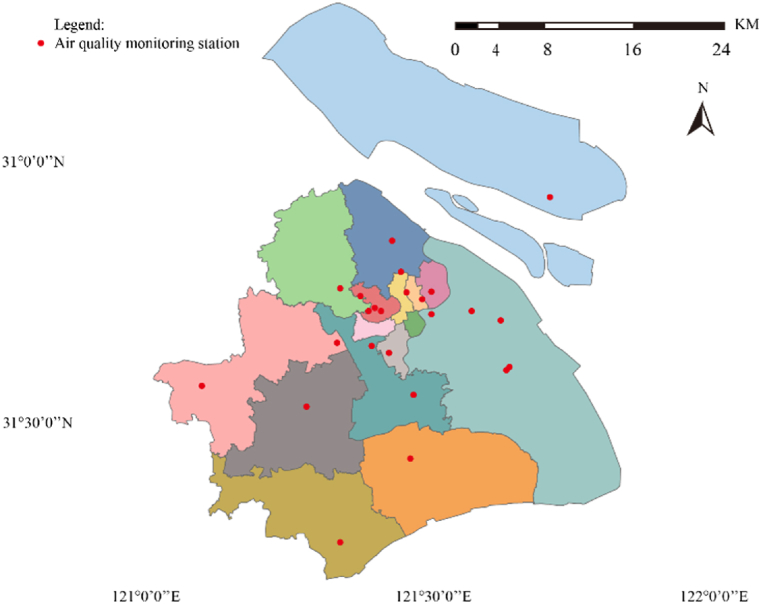
Distribution of air quality monitoring stations (only) in Shanghai.

In this paper, the data of 19 environmental quality monitoring stations in Shanghai is selected for analysis. Shanghai is located in the Yangtze River Delta region along the east coast of China with small topographic relief. It is a typical megacity with an area of about 6340 km^2^ and has a subtropical monsoon climate. The air quality monitoring stations in China and Shanghai are shown in Fig. 5.

The whole city is divided according to the administrative boundaries, and the meteorological data obtained are rasterized by inverse distance weighting method. Then the data will be projected and unfolded at each point. After that, the hourly concentrations of other pollutants and meteorological data are converted into daily data.

Shanghai is one of the most populous cities in the world and a major financial hub in China. As of 2022, it had a population density of around 3800 people per square kilometer. The city's population density is relatively high due to its status as a global economic center and its attractiveness for job opportunities, which leads to a continuous influx of people from various regions within China and abroad.

Shanghai is known for its heavy traffic congestion, especially during rush hours. The city's extensive network of roads, bridges, tunnels, and public transportation systems experiences high traffic volumes due to the large population and significant economic activities.

Air pollution in Shanghai is mainly attributed to several factors, including industrial activities, vehicular emissions, construction dust, and power generation. Industrial facilities, especially those involved in manufacturing and energy production, release a considerable level of pollutants into the air. Vehicular emissions, especially from the ever-increasing number of vehicles in the city, also contribute to the high levels of air pollution. Construction activities, common in a rapidly developing city like Shanghai, generate large amounts of dust particles that contribute to the city's air pollution levels.

PM_10_ and PM_2.5_ mainly originate from the combustion of fossil fuels, industrial processes, and dust from construction activities and road traffic.

#### 2.3.2 Data acquisition and preparation

The dataset in this paper can be obtained from the website by using python. The data can be divided into 3 categories: concentrations of other pollutants, meteorological data, and macro variables. Specifically, the concentrations of other pollutants (AQI, SO_2_, CO, PM_10_, O_3_, and NO_2_) can be obtained from Zhenqi.com (https://www.zq12369.com). The meteorological data (2013–2021) includes the sunshine duration, humidity and wind speed can be obtained from China Meteorological Administration (http://www.cma.gov.cn), and the precipitation data (2013–2021) is available from European Centre for Medium-Range Weather Forecasts (https://www.ecmwf.int/), and the temperature data (2013–2021) from the National Oceanic and Atmospheric Administration (https://www.noaa.gov). The macro variables (population density, car ownership, coal use, sunny days, etc.) can be obtained from the statistical yearbook of Shanghai over the years. Other pollutant concentrations and meteorological data include daily and hourly data, macro variables are annual data, and car ownership and electricity consumption are monthly data. The higher the proportion of coal in energy consumption, the more serious the air pollution caused by the secondary industry. What's more, the correlation between coal and energy consumption is lower than 10 % showing that it’s necessary to take both variables into consideration. While GDP are important indicators of the economy, we find that the high correlation between GDP, population density and private car ownership could bring multicollinearity (month by month data) which will lead to the failure of significant test when analyzing the relationship of PM_2.5_ and these 3 variables.

The variables and symbols involved in the empirical analysis of this paper are listed in Table 1.

**Table 1:** Variables and symbols involved in this paper.

Index	Variable name	Symbol
Air pollutant indicators (Type I)	Inhalable micro particulate matter	PM_2.5_
	Inhalable particulate matter	PM_10_
	Carbon monoxide	CO
	Nitrogen dioxide	NO_2_
	Sulfur dioxide	SO_2_
	Ozone	O_3_
Meteorological environmental indicators (Type II)	Precipitation	X_1_
	Sunshine duration	X_2_
	Humidity	X_3_
	Wind speed	X_4_
	Average temperature	X_5_
	Evaporation	X_6_
Macroeconomic indicators (Type III)	Private car ownership	Cars
	Population density	density_of_people
	Annual coal use	coal_amount
	Energy loss	loss_of_energy
	Number of sunny days	number_of_good_weather
	Air quality rate	rate_of_excellent_whethe
	Energy consumption	Energy
	Total population	people

The calculation of the average daily PM_2.5_ concentration in the city is based on the *Technical Specifications for Ambient Air Quality Assessment (Trial)* issued by the Ministry of Environmental Protection [35]. The daily average concentration of PM_2.5_ at an observation station is the arithmetic mean of the hourly PM_2.5_ concentration of the station from 0:00 to 24:00 every day. The proportion of missing values in the observed PM_2.5_ concentration data of all observation stations in the city at a certain time t is calculated. When the proportion is less than 25 %, the arithmetic mean of all observation stations is the PM_2.5_ concentration in Shanghai at that time. When there are many missing data, that is, the proportion is ≥ 25 %, the maximum among all observed values is taken as the PM_2.5_ concentration in Shanghai [21]. For example, Jung et al. [36] adopted this method when dealing with the missing PM_2.5_ concentration values in Japan, and achieved good results.

## 3 Empirical analysis

### 3.1 Screening of important stations in Shanghai

A hierarchical analysis method was applied by Liang et al. [37] for an index system in evaluating environmental pollution levels in an urbanization process. As based on entropy weight method and principle of minimum information entropy, such method has an important reference in determining the impact weights of different regions. The 19 observation stations in Shanghai are scored by entropy weight. Let *X*_*ij*_ be the *j*-th evaluation index of the *i*-th observation station, and *Y*_*ij*_ be the index after standardization. $P_{ij}$ is the proportion of the *j*-th pollution gas to the *i*-th observation station, $E_{j}$ is the entropy of each indicator, $W_{j}$ is the weight of each indicator, and $Z_{i}$ is the score of each station.

(30) $$\begin{array}{ccc} P_{ij}=\frac{X_{ij}}{\sum_{i=1}^{m}X_{ij}}\text{,} & i=1,2\text{,}\ldots ,m\text{,} & j=1,2\text{,}\ldots ,n \end{array}$$

(31) $$\begin{array}{cc} E_{j}=-\frac{1}{\ln\left(m\right)}\cdot \sum_{i=1}^{m}P_{ij}\cdot \ln\left(P_{ij}\right)\text{,} & j=1,2\text{,}\ldots ,n \end{array}$$

(32) $$\begin{array}{cc} W_{j}=\frac{1-E_{j}}{\sum_{i=1}^{m}\left(1-E_{j}\right)}\text{,} & j=1,2\text{,}\ldots ,n \end{array}$$

(33) $$\begin{array}{cc} Z_{i}=\sum_{j=1}^{n}W_{j}\cdot y_{ij}\text{,} & j=1,2\text{,}\ldots ,n \end{array}$$

The score $Z_{i}$ of each station is converted, ranging between 60 and 100. The higher the score, the larger the pollution index. The results of pollution level are shown in Table 2.

**Table 2:** Air quality results evaluation at 19 observation stations in Shanghai.

Station name^a^	PM_2.5_	Industrial waste gas (sulfur nitride)	Others	Comprehensive index of air quality	Score
Yangpu Sipiao	30.360	30.546	20.199	18.895	100.000
Jing'an Observation Station	31.438	23.597	20.606	19.431	95.072
Putuo	30.657	25.038	19.455	19.197	94.347
Hongkou	28.913	29.067	18.447	17.769	94.195
Shanghai Normal University, Xuhui Campus	29.853	25.824	20.023	17.977	93.676
Nanxiang Town, Jiading	26.897	28.635	17.742	17.036	90.310
Miaohang Town, Baoshan	30.349	22.287	17.467	19.799	89.902
Shiwuchang Station	29.357	21.170	20.646	17.665	88.837
Zhangjiang, Pudong	28.736	22.821	18.706	17.422	87.684
Pudong New Area Observation Station	27.957	22.795	19.298	17.500	87.550
Xujing Town, Qingpu	29.559	19.377	17.641	18.809	85.385
Chuansha Town, Pudong	26.866	17.124	19.228	16.799	80.017
Songjiang Library	27.997	16.143	18.005	17.750	79.895
Xianxia Street, Changning	29.499	13.060	17.606	19.062	79.228
Huinan Town, Pudong	25.402	14.733	18.407	17.102	75.645
Pujiang Town, Minhang	28.134	10.720	17.722	17.949	74.525
Fengxian Nanqiao New City	26.759	9.858	19.013	17.952	73.581
Jinshan New Town	26.662	1.685	19.501	17.500	65.348
Dongtan School Affiliated to Shanghai Experimental School, Chongming	22.297	5.417	19.057	15.186	61.956

a The evaluation ranking is sorted by score of each observation station. Higher scores indicate worse air quality.

It can be seen from Table 2 that the heavily polluted areas are concentrated in Yangpu, Jingan, Hongkou, Xuhui and other urban areas, while Qingpu, Pudong, Songjiang, Jinshan, and Fengxian – districts located in the suburbs – are less polluted, with relatively good air quality. Among the five new urban districts, Jiading has slightly worse air quality, which is related to the proportion of industry and population density.

According to the PM_2.5_ concentration, the pollution can be divided into three levels: severe pollution, mild pollution, and light pollution, and the corresponding score ranges of the station are 90–100, 80–90, and 60–80, respectively. To ensure selected stations are representative, after the importance of each station is calculated by XGBoost model, the top two sites with the highest importance are selected respectively from the three pollution levels. It should be pointed out that the importance of the low pollution group is generally low, so only Minhang station is retained. Finally, the five most important observation stations, Pudong (Huinan Town), Jiading (Nanxiang Town), Qingpu (Dianshan Lake), Hongkou and Minhang (Pujiang Town) are selected to construct the weight matrix.

### 3.2 Analysis of the importance of meteorological variables

**Fig. 6:**
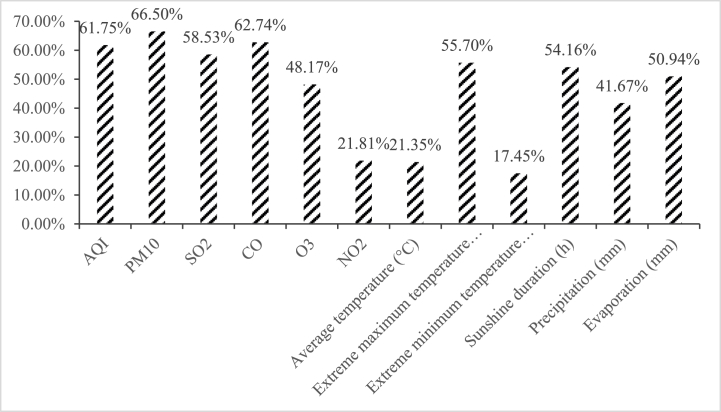
Importance ranking of environmental factors affecting PM_2.5_ concentration.

All matched variables are independent variables, PM_2.5_ concentration is the dependent variable. The importance of independent variables is ranked by XGBoost and the results are shown in Fig. 6. Generally, when the importance of a variable is lower than 20 %, the variable is considered to have a weak correlation with PM_2.5_, so the variable should be eliminated. Next, stepwise regression is performed on the remaining variables that have passed the importance screening, and variables that fail the significance test are removed.

For annual data such as cumulative precipitation, evaporation, and number of sunny days, it is necessary to convert them into monthly data according to frequency, and then match them with the monthly average PM_2.5_ concentration to expand the sample size, and further study their relationship with PM_2.5_. Herein, the start time of 2003 is *t* = 1, and the end time of 2021 is *t* = 18. Each year is an interval, and the cubic spline interpolation in Eq. (34) is used for data expansion. The value of $\alpha_{ij}$ is determined by the chosen fitting function and can be given by Eviews fitting

(34) $$f\left(t\right)=\alpha_{1i}+\alpha_{2i}\cdot \left(t-t_{i}\right)+\alpha_{3i}\cdot {\left(t-t_{i}\right)}^{2}+\alpha_{4i}\cdot {\left(t-t_{i}\right)}^{3}$$

where $t_{i}\leq t\leq t_{i+1}$ and *i* = 1, 2, *N*−1.

There is a lag correlation between some variables, such as air temperature and wind speed, and PM_2.5_. For these data, we first substitute them into Eq. (35), and the lag period is determined by multiple regression. Then Eq. (34) is applied to calculate the correlation of each variable with PM_2.5_.

(35) $${PM}_{2.5}=\beta_{0}+\beta_{1}\cdot A_{t-i}+\beta_{2}\cdot B_{t}$$

where, $A_{t-i}={\left(X_{1},X_{2},X_{3},\ldots \right)}^{T}$ represents the column vector composed of variables with lag compared with PM2.5; $B_{t}={\left(B_{1},B_{2},B_{3},\ldots \right)}^{T}$ represents the column vector composed of variables with synchronization compared with PM_2.5_; and $\beta_{1}=\left(\alpha_{1},\alpha_{2},\alpha_{3},\ldots \right)$ and $\beta_{2}=\left(\gamma_{1},\gamma_{2},\gamma_{3},\ldots \right)$ are the coefficient matrices of each independent variable.

(36) $$VIF=\frac{1}{1-R^{2}}$$

Forming together vectors $A_{t-i}$ and $B_{t}$ retrieved from Eq. (35), we obtain the matrix $X=\left[\begin{array}{cc} A_{t-i} & B_{t} \end{array}\right]$ and calculate the eigenvalues of the matrix $X^{T}X$. The smaller the absolute value of the eigenvalues of a variable, the higher possibility of multicollinearity between this variable with other independent variables would be. Using Eq. (36), we can then determine whether the variable should be retained.

After frequency conversion, the importance of variables is calculated, as shown in Fig. 6

Among the variables in Fig. 6, extreme minimum temperature fails the significance test, and other variables are finally determined as independent variables. The importance of independent variables is shown as in Fig. 6. Among all indicators, the PM_10_ concentration ranks first in importance for predicting PM_2.5_ concentration, with a correlation of 66.5 %, and AQI, CO and SO_2_ has a correlation of about 60 %. Precipitation and monthly evaporation have a great impact (negative) on PM_2.5_ concentration. The reason is that the increase in air humidity will take away part of the pollutants and cause part of the gas particles to settle, which is conducive to improving air quality.

### 3.3 Analysis of factors affecting PM_2.5_

#### 3.3.1 Ranking of the importance of factors affecting PM_2.5_

The correlation between each variable and PM_2.5_ is determined according to the XGBoost importance score (AUC value). Its disadvantage is that dependent variables need to be transformed into categorical variables before screening. Note that the data collection frequency of macroeconomic factors cannot be measured in terms of days and thus needs to be converted by Eq. (34). Therefore, compared with meteorological factors, the sample size of macroeconomic counterparts is relatively small. We notice the effectiveness of grey correlation analysis in this case, as can solve the probable error caused by insufficient sample size, and is more helpful to explore the relationship between macroeconomic factors and PM_2.5_. Therefore, Eqs. (37), (38) can be used to investigate the relationship of each time series. This method considers the sequence of each element and can make up for the error caused by the absence of sequence.

(37) $$\varepsilon_{i}=\frac{\underset{i}{\underbrace{\min}}\underset{k}{\underbrace{\min}}\left|X_{0}\left(k\right)-X_{i}\left(k\right)\right|+\rho \cdot \underset{i}{\underbrace{\max}}\underset{k}{\underbrace{\max}}|X_{0}\left(k\right)-X_{i}\left(k\right)|}{\left|X_{0}\left(k\right)-X_{i}\left(k\right)\right|+\rho \cdot \underset{i}{\underbrace{\max}}\underset{k}{\underbrace{\max}}|X_{0}\left(k\right)-X_{i}\left(k\right)|}$$

(38) $$r_{i}=\frac{\sum_{k=1}^{n}\varepsilon_{i}\left(k\right)}{n}$$

where ρ is the resolution coefficient, which it is set to 0.5, the commonly used value in literature; $\varepsilon_{i}$ is the grey correlation coefficient between each variable and PM_2.5_ concentration at a certain time; $\underset{i}{\underbrace{\min}}\underset{k}{\underbrace{\min}}\left|X_{0}\left(k\right)-X_{i}\left(k\right)\right|$ and $\underset{i}{\underbrace{\max}}\underset{k}{\underbrace{\max}}|X_{0}\left(k\right)-X_{i}\left(k\right)|$ are the minimum and maximum differences; $r_{i}$ is the grey correlation index between the independent variable and PM_2.5_ concentration.

**Fig. 7:**
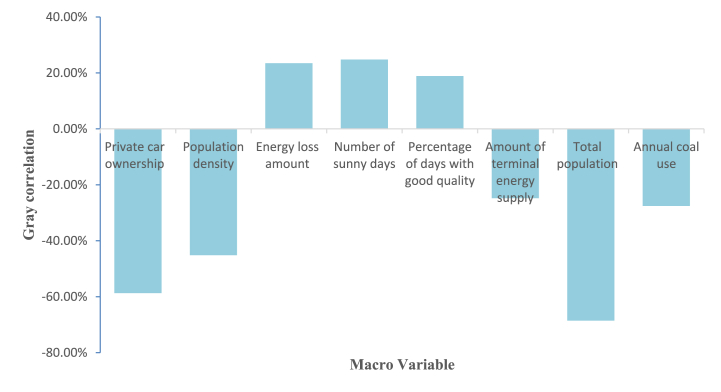
Importance of economic factors.

The frequency conversion of macro variables uses Eq. (34), and the conversion function is the quadratic interpolation curve in the Eviews (this function can be modified until the fitting error is minimal). For data such as population density and industrial structure distribution in Shanghai, the fluctuation of data is not obvious after changing to daily data, so the constant method is adopted for conversion. After conversion, Fig. 7 can be obtained by Eqs. (37), (38).

It can be seen from Fig. 7 that the private car ownership, population density, coal consumption, and total population in Shanghai are negatively correlated with PM_2.5_ concentration, and the correlation coefficients are −58.7 %, −45.15 %, −27.55 %, and −68.52 %. Among them, private car ownership and total population is highly correlated with PM_2.5_ concentration, and their correlation coefficients both exceed 55 %. In recent years, the PM_2.5_ concentration has not shown a fluctuating upward trend with the popularization of private cars and the massive influx of population in Shanghai. The number of days with severe pollution was 46 between 2013 and 2015, while the total number of days with severe pollution in the six years from 2016 to 2021 was less than 40, indicating that Shanghai's air quality is improving. Due to Shanghai's special license plate policy, the choice of new energy vehicles is a favorable means for migrants to obtain license plates, resulting in the growth of private cars mainly concentrated in the new energy field, which explains the negative correlation. In addition, Pudong, Songjiang and other new areas are developing high-tech industries, and the secondary industry is also moving to the surrounding areas of Shanghai. Such factors are possible influencing factors of PM_2.5_ concentration.

#### 3.3.2 Prediction after introduction of different variables

This section will discuss whether to add these three types of variables (air pollutant indicators, meteorological environmental indicators, and macroeconomic indicators), and compare the prediction accuracy and corresponding error of the XGBoost model before and after introduction of different independent variables, as shown in Table 3.

**Table 3:** Prediction results comparison after variables are added.

Variable added	Group	MAE	Goodness of fit (*R*^2^)	RMSE	RPE
Type I	Trained	0.8295	99.08 %	1.179	2.11 %
	Test	3.7490	89.45 %	4.898	9.05 %
Types I and II	Trained	0.7796	99.13 %	1.149	2.06 %
	Test	3.6735	89.89 %	4.801	8.86 %
Types I and III	Trained	0.7980	99.14 %	1.140	2.04 %
	Test	3.7737	89.32 %	4.927	9.11 %
Types I, II, and III	Trained	0.7726	99.17 %	1.122	2.01 %
	Test	3.8586	88.60 %	5.091	9.41 %

MAE: Mean absolute prediction error; RMSE: Root mean square error; RPE: Relative prediction error.

After introduction of variables of type II, the average decision error and root mean square error of the model are slightly reduced and the goodness of fit is slightly improved on both the training set and the test set. The only environmental variable that has a greater impact on PM_2.5_ concentration is rainfall. After introduction of environmental variables, the error term is improved by less than 5 %, and the goodness of fit is not significantly enhanced. The goodness of fit does not decrease after removing the environmental variables with low correlation such as the highest temperature, lowest temperature and sunshine duration, so they can be eliminated.

Also shown in Table 2, the performance of the model on the training set is improved to a certain extent after the introduction of the processed type I variables. Moreover, the goodness of fit is increased by about 1 %, and the relative error is reduced. By contrast, on the test set, the effect of these four evaluation indicators decline.

Similarly, when the three types of variables are introduced at the same time, the evaluation indices on the training set are further improved. However, on the test set, the relative prediction error increases by 0.36 % and the mean square error drops by 2.28 %. When only precipitation is added to the model, the error on the test set and the training set are reduced, and the performance on the training set and the testing set is consistent. It is therefore can be concluded that the prediction accuracy of the XGBoost model cannot be improved by simply increasing the number of variables.

### 3.4 Empirical results

#### 3.4.1 Signal decomposition results

**Fig. 8:**
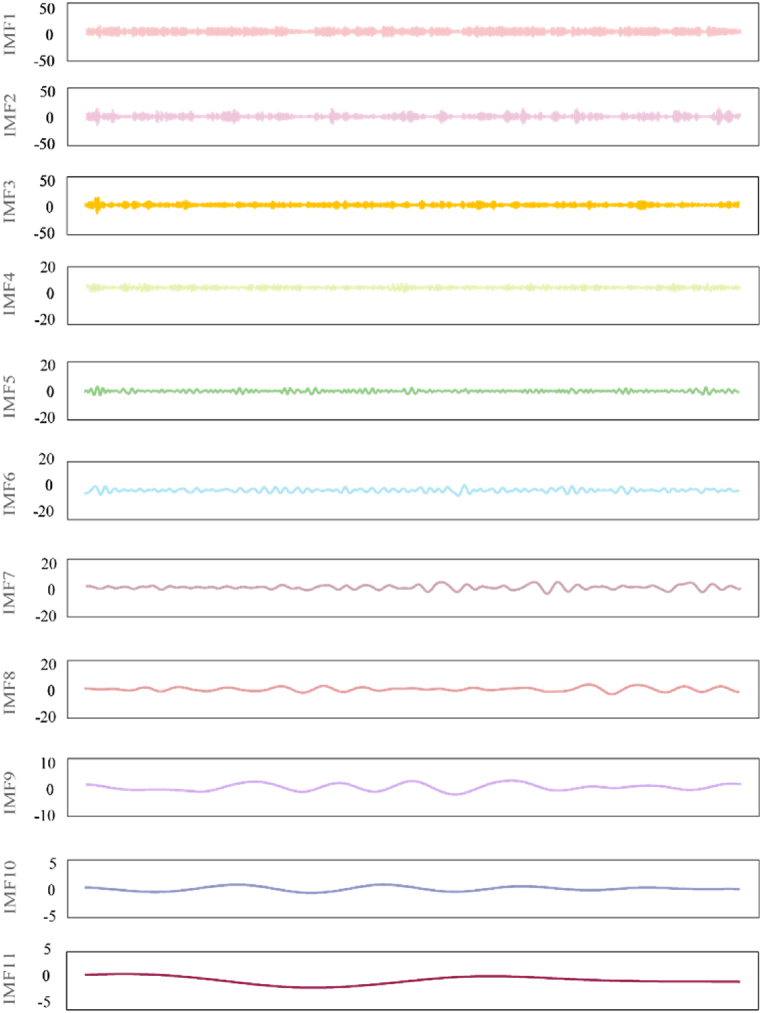
Sequence decomposition effects using EEMD method.

The EEMD model is used to decompose the PM_2.5_ sequence, and the results are shown in Fig. 8.

Among the 11 subsequences decomposed, the first five IMFs are high-frequency sequences, the 6–11 sequences are low-frequency sequences, and the last item is the residual term. Generally, when the number of integrations is set to around 100 and the original sequence is processed with white noise, the decomposition effect can be improved and the number of iterations required for convergence can be effectively reduced. First, the mean square error of the original sequence is calculated, and then the variance of the white noise is obtained by introducing the coefficient factor for correction. Then, white noise is added to the original sequence to get a new sequence. It is found that when the standard deviation of white noise is 0.1, the effect is better. Therefore, noise_width is set to 0.1, which is added to the 11 IMF subsequences and the 1 residual sequence obtained after decomposition. The residual sequence shows the original variation trend of the daily average concentration of PM_2.5_ in Shanghai. The characteristics of the decomposed subsequences are shown in Table 4.

**Table 4:** Characteristics of decomposed sequences using EEMD method.

Sequence	Index						
	Kendall's correlation coefficient	Variance contribution rate	Pearson's correlation coefficient	Spearman's correlation coefficient	Period (day)	Sample entropy	Number of run
IMF1	0.5280	48.34 %	0.7326	0.7239	1.4309	1.2538	1496
IMF2	0.3243	8.13 %	0.4609	0.4704	2.5209	0.4688	1114
IMF3	0.2960	4.75 %	0.4215	0.4350	3.5185	0.8775	760
IMF4	0.2405	4.63 %	0.3505	0.3556	6.2147	0.6269	431
IMF5	0.1926	3.52 %	0.2789	0.2854	11.4419	0.5679	244
IMF6	0.1527	1.7 %	0.2322	0.2275	21.2374	0.4532	127
IMF7	0.1733	2.61 %	0.2559	0.2528	42.1714	0.1868	60
IMF8	0.1593	2.35 %	0.2378	0.2362	86.8235	0.0994	32
IMF9	0.0967	1.19 %	0.1518	0.1444	184.5	0.0413	28
IMF10	0.0675	0.5 %	0.0989	0.0994	295.2	0.0263	10
IMF11	0.0369	0.43 %	0.0641	0.5513	984	0.0069	4
R	0.0256	0.58 %	0.0485	0.0373	2952	1.1324 × 10^−4^	2

From the decomposed sequence, it can be seen that from IMF1 to IMF11, the frequency of subsequences decreases and the number of periods increases. The average number of periods per sequence is obtained by dividing the total number of observation days by the number of extreme points. The average period of IMF3 is 6.2147 days, which is about one week; the period of IMF5 is 21.2374 days, more than half a month; the period of IMF6 is 42.1714 days, which is one and a half months; the average period of IMF8 is 184.5 days, which is about half a year. It can be seen that the PM_2.5_ concentration in Shanghai changes roughly on a weekly, monthly and quarterly basis.

IMF1 is the most important subsequence with a variance contribution rate of 48.34 %. The variance contribution rates of IMF2–IMF4 are 8.13 %, 4.75 %, 6.63 % and 3.52 %, respectively. The rest of the subsequences have low contribution rates. The cumulative variance contribution rate of the first five items reaches 69.37 %, nearly 70 %. The variance contribution rate of all subsequences is 78.73 %. Overall, IMF1–IMF4 have strong correlation with the original sequence.

The mean of each subsequence is calculated respectively. The data points less than the mean in each subsequence are denoted as “−”, and the points larger than the mean are denoted as “+”. Therefore, 12 symbol sequences (11 components and a residual vector) can be obtained. Then, the number of runs of the sequence is calculated. The larger the number of runs, the stronger the randomness and fluctuation of the sequence. It can be found that the number of runs of the sequence plunges from IMF1 to R. The “run_test” proves that all subsequences pass the run test. The time span of the original PM_2.5_ sequence is 2587 days, and the sample entropy of each subsequence (including the original sequence) is calculated. In general, the tolerance for a sequence should be set to 0.2 times its standard deviation.

Before reconstruction, the PE of subsequence should be calculated. After comparison, when the dimension of embedding number is set to 5 and the time delay parameter τ to 1, the PE of each component is 0.913, 0.457, 0.456, 0.358, 0.173, 0.163, 0.137, 0.108, 0.008, 0.006, 0.001, and 0 respectively. Thus, components 1–4 are reconstructed as high-frequency components, 5–7 as low-frequency components, and 8–11 as residual components, and the PE of residual R is 0. Table 5 presents the sequence prediction results after reconstruction.

**Table 5:** Sequence prediction results after reconstruction.

Sequence	Group	MAE	Goodness of fit (*R*^2^)	RMSE	RPE
Reconstructed PM_2.5_ sequence	–	0.745	85.74 %	0.929	8.80 %
high_fre	Trained	0.744	95.24 %	0.920	1.65 %
	Test	1.147	88.70 %	1.418	12.1 %
low_fre	Trained	0.710	90.32 %	0.9160	20.07 %
	Test	0.866	85.6 %	1.117	29.63 %
R	Trained	1.297	95.93 %	1.634	50.66 %
	Test	5.495	38.38 %	6.324	40.34 %

MAE: Mean absolute prediction error; RMSE: Root mean square error; RPE: Relative prediction error.

The reconstruction process follows the steps of “reconstruction by frequency, first prediction, and then superimposition”. Herein, high_fre, low_fre and R represent high frequency sequence, low frequency sequence and residual sequence respectively. For the original daily data PM_2.5_ concentration, the correlation coefficient between the decomposed subsequence and the original sequence is calculated. The sequence whose absolute value of correlation coefficient is greater than 0.2 is selected for reconstruction according to the frequency. The subsequence is considered to contain less information of the original sequence when its correlation coefficient is less than 0.2, so the sequence is not involved in reconstruction. Three reconstructed sub-sequences are obtained: high-frequency subsequence, low-frequency subsequence, and residual sequence. XGBoost prediction is performed on these three subsequences. On the training set, the goodness of fit of the residual sequence is the best, and that of low-frequency sequences and high-frequency sequences can reach about 90 %. On the test set, the goodness of fit of low-frequency sequences and high-frequency sequences is about 85 %, but that of the residual sequence is low, only 38.38 %. This is reasonable since the residual sequence itself only contains a little short-term fluctuation information of the original sequence. On the test set, the relative prediction errors of high-frequency sequences, low-frequency sequences, and residual sequences are 12 %, 38 %, and 40 %, respectively, but the low-frequency sequence has the largest mean absolute error of 6.7 %.

**Fig. 9:**
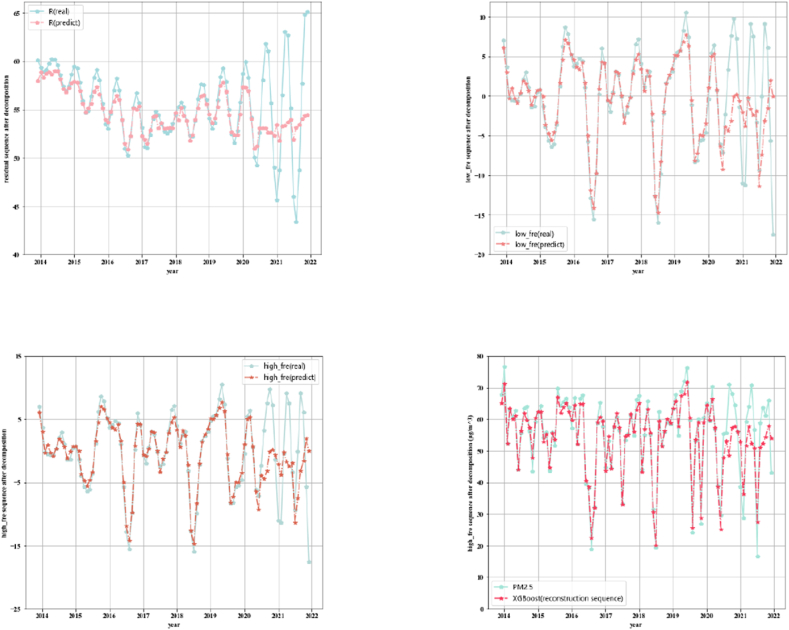
PM2.5 concentration predicting results generated by sub-sequences of different frequencies after first prediction and sequence reconstruction.

Next, these three subsequences are superimposed and compared with the original PM_2.5_ sequence. The mean absolute error of the reconstructed sequence is 0.745, the root mean square error is only 0.929, and the relative prediction error is 8.8 %. These indicate that the reconstructed sequences well reflect the inherent fluctuations and changes of the PM_2.5_ sequence, and achieve the purpose of reducing the prediction error of the XGBoost model through signal decomposition. The predictions of the high-frequency sequence, the low-frequency sequence, and the residual sequence are compared to obtain the reconstructed prediction sequence of PM_2.5_ concentration. The comparison of the prediction curve on the test set with the original curve is shown in Fig. 9.

The reconstructed sequence has poorer prediction performance in the three periods of mid-August 2020, late November 2020, and late December 2020, when PM_2.5_ concentration increased significantly (intensified pollution), and the prediction effect of the remaining periods all reaches the expectation. The wild fluctuations in PM_2.5_ since 2020 also reflect the impact of urban development on environment in recent years. This method of selecting the subsequences with high correlation for first reconstruction and then performing the second reconstruction after prediction is an “incomplete reconstruction” at the cost of partial information. If all subsequences are allowed to participate in the reconstruction, it is difficult to avoid the problem of “modal aliasing”.

#### 3.4.2 Prediction results of modified spatiotemporal XGBoost model

The three methods of XGBoost, LightGBM, and GBDT after parameter adjustment and signal decomposition are used as the first layer of the integrated model (these three methods are all gradient boosting algorithms, which are conducive to model fusion). The error on the test set of the training set is calculated respectively, and the average of the three prediction sequences is calculated to obtain the first prediction sequence. In order to avoid overfitting, the second layer model should not be complicated. The purpose of adding the second layer model is to make the trend extrapolation of the fused model easier. Since the second layer model conducts is the second prediction based on the first layer of prediction sequence, the prediction accuracy will be reduced if the second layer model is complex. Therefore, a simple linear regression model is selected for prediction, and the final fusion model obtained is called the spatiotemporal XGBoost model. Comparison of prediction results among the three models is shown in Table 6.

**Table 6:** Errors of stacking fusion models.

Model	Group	MAE	Goodness of fit (*R*^2^)	RMSE	RPE
First layer: LightBGM	Trained	3.99	96.67 %	6.49	10.90 %
	Test	5.8041	92.51 %	8.53	16 %
First layer: XGBoost	Trained	1.9775	99.41 %	2.535	4.92 %
	Test	3.8256	98.07 %	4.94	8.03 %
First layer: GBDT	Trained	2.7400	98.12 %	3.54	6.36 %
	Test	5.7336	95.04 %	7.69	14.92 %
Second layer: Multiple regression	–	3.3485	98.10 %	6.59 %	9.06 %

MAE: Mean absolute prediction error; RMSE: Root mean square error; RPE: Relative prediction error.

**Fig. 10:**
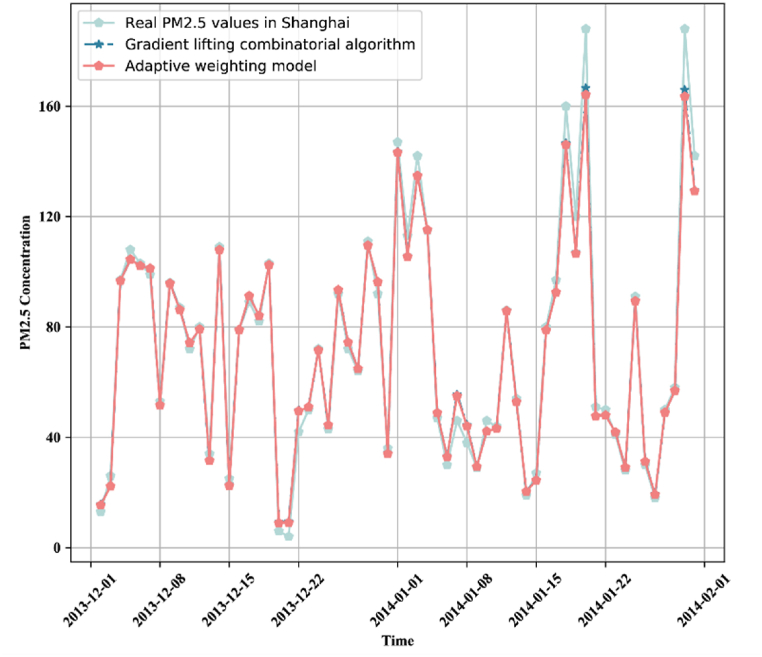
Accuracy comparison of adaptive weighting method against the traditional method.

It can be seen from Table 6 that the prediction accuracy of the fusion model is around 90 %. The 8 variables with the strongest correlation with PM_2.5_ concentration are used as independent variables, and the high-frequency, low-frequency, and residual sequences reconstructed after signal decomposition as dependent variables. XGBoost is used for prediction, and then the prediction sequence is superimposed to obtain the prediction result of XGBoost in the first layer of the fusion model. Similarly, the above operations are performed with LightBGM and GBDT respectively to obtain their prediction sequences. As shown in Table 6, the goodness of fit of each layer model is above 90 %. The mean absolute error of the fusion model is 3.3485, and the goodness of fit is 98.1 %, which is significantly improved compared with the single model. The performance of XGBOOST processed by the adaptive weighting method on the test set is shown in Fig. 10.

**Fig. 11:**
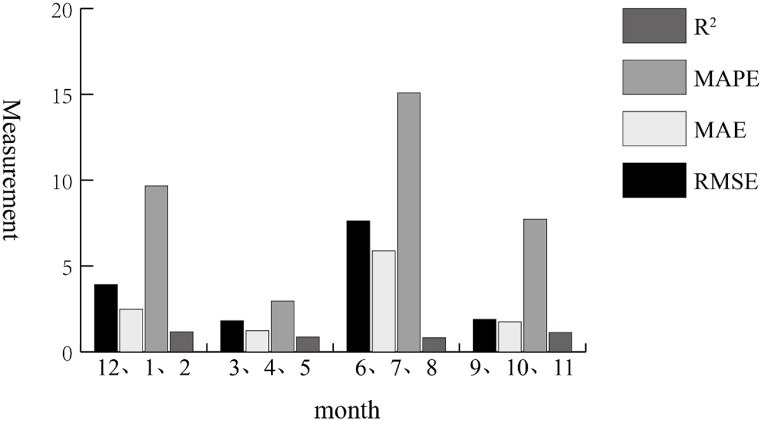
XGBoost prediction results for different seasons.

The goodness of fit of the predicted value and the actual value regression of the Stacking fusion model, XGBoost, LightBGM, GBDT, and the XGBOOST model modified by adaptive weighted method are 0.977, 0.993, 0.989, 0.988, and 0.993, respectively. This indicates that the adaptive weighting method improves the goodness of fit and reduces the prediction error. It can be seen from Fig. 11 that the performance of the model at extreme points is also improved. The fitting equation between the actual value and the predicted value of the final fusion model is

(39) y=1.026·x−1.548

The spatiotemporal XGBoost model is modified by the adaptive weight adjustment method. The fitting equation between the predicted value of the modified model and the actual value is *y* = 0.9709·*x* + 1.4527 (Eq. (39) is the fitting equation before the model modification). *R*^2^ is 0.993, which achieves the desired effect. Finally, the established model is used to forecast the PM_2.5_ sequence in Shanghai, and Fig. 11 is obtained.

In the 10-year observation data, March, April, and May represent spring; June, July, and August represent summer; September, October, and November represent autumn; December, January, and February represent winter. The XGBoost model after parameter adjustment has better predictions in spring and autumn. The possible reason is that the pollution is more serious in winter and there are many extreme points of PM_2.5_ concentration, which impact the prediction accuracy. The error of each evaluation indicator in April (spring) is far smaller than that in other seasons. The active atmosphere in summer leads to the accelerated migration rate of air pollutant particles, so it is not easy for more PM_2.5_ particles to accumulate in local areas. The prediction performance of the XGBoost model differs in different seasons, which also reflects the difference in meteorological conditions among seasons.

#### 3.4.3 Model performance after introduction of observations from each station

**Fig. 12:**
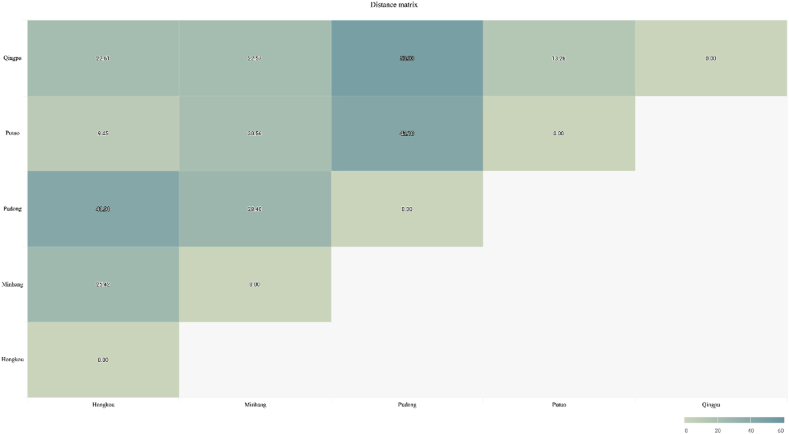
The heatmap of distance matrix between every two of the top five observation stations of highest correlation with each other.

The surface arc length distance *r* between two places is obtained. One observation station is reserved in each administrative region, so there are a total of 12 observation stations in Shanghai, as shown in Fig. 12.

The top 5 observation stations of XGBoost importance are selected: Hongkou, Minhang (Pujiang), Pudong (Huinan), Putuo and Hongkou, which are used as distance matrix to participate in weighting. The mutual influencing factors of stations are introduced for secondary prediction. There are two processing methods for the distance between two stations, namely the inverse distance weighting method and the bandwidth selection method. The empirical analysis results of these two distance processing methods are given. The optimal bandwidth combination of each station is selected, and the mean square error of the observation station under the bandwidth is calculated with the weight. The optimal bandwidths of Fengxian, Pudong New Area, Putuo, Yangpu and Xujiahui are 42 km, 41 km, 22 km, 8 km and 62 km respectively. The corresponding root mean square errors are 16.1453, 16.8092, 16.8145, 17.2938, and 17.4082, respectively.

### 3.5 Extending XGBoost's spatial prediction by kriging interpolation

**Fig. 13:**
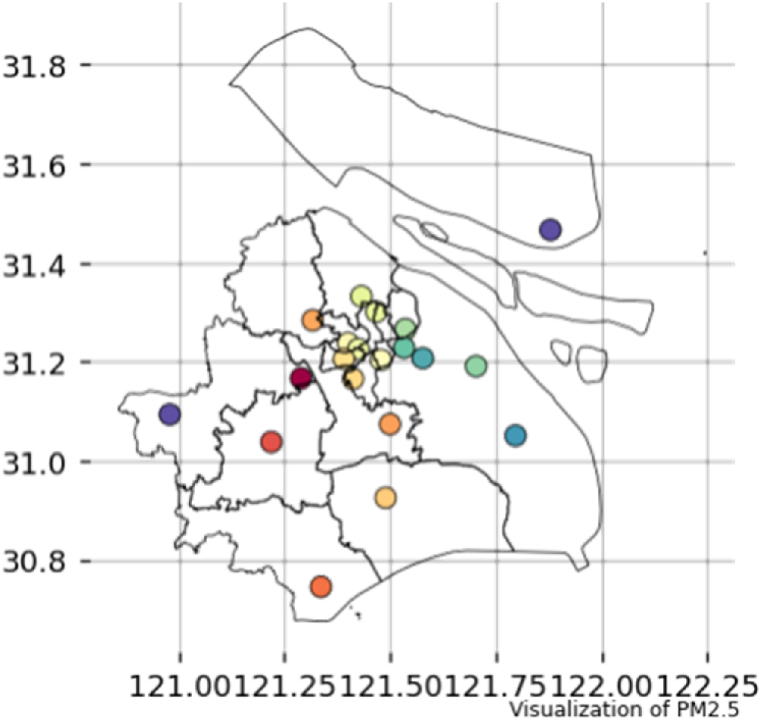
Distribution of PM_2.5_ concentration (μg/m^3^) in each district in Shanghai.

The modified XGBoost model is used to predict the PM_2.5_ concentration of 19 stations in Shanghai. The predicted result series filled with missing values are summarized to calculate the annual PM_2.5_ concentration of each station. The time span of the predicted result is from 1/1 to 12/31 in 2021. After inverse distance weighting correction, the distribution of PM_2.5_ concentration in Shanghai is obtained, as shown in Fig. 13.

**Fig. 14:**
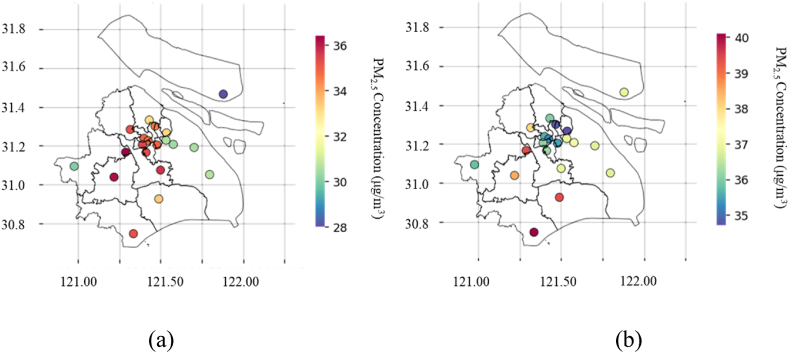
Distribution of PM_2.5_ concentration (μg/m^3^) in each district in Shanghai by time: In summer (a) and in winter (b), respectively.

**Fig. 15:**
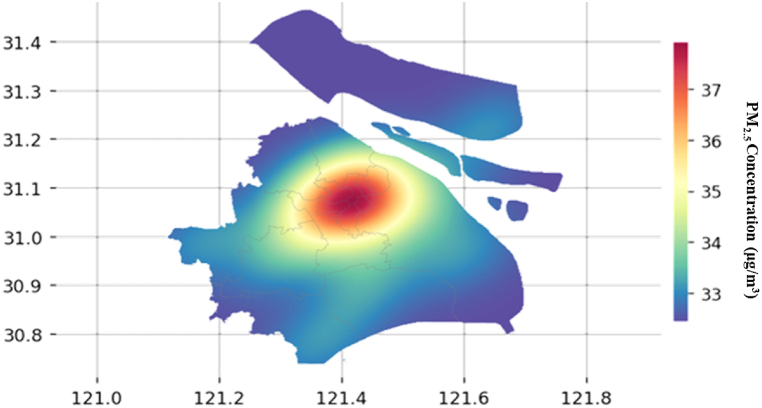
Distribution of PM_2.5_ concentration predicted values (μg/m^3^) of across Shanghai, using Kriging interpolation method.

It can be seen that the PM_2.5_ concentration in Putuo, Xujiahui, Yangpu and other urban areas is at a high level, especially in the Yangpu Industrial Zone. The distribution of PM_2.5_ concentration is quite similar to that of industrial areas and population density. The accuracy of spatial prediction is consistent with the traditional understanding of air pollution distribution. Seen from Fig. 15, the PM_2.5_ concentration in winter is at a high level, and air pollution is more serious, which may be resulted from the climatic characteristics of winter. The frequent rainfall in summer is an important reason for the improvement of air pollution, as shown in Fig. 14.

Next, Kriging interpolation method is used to establish a spatial distribution network for the predicted values of each observation station. And the kernel density function is a Gaussian function. When the Kriging conditions are met, the PM_2.5_ concentration value of the adjacent station is filled in. During the filling process, the actual value of the observation station is used for training and correction to ensure the accuracy of the established spatial PM_2.5_ network. Finally, by reading the boundary data (.json file) of each administrative district in Shanghai, the spatial PM_2.5_ gridded data is cut and divided, and the predicted PM_2.5_ concentration of each station is obtained, as shown in Fig. 15.

It is further confirmed in Fig. 15 that the areas with the most serious air pollution in Shanghai are the old urban areas and industrial areas. In urban areas, PM_2.5_ concentration is on the decline as it approaches to suburban areas. As for the real-time evaluation system for the observation stations, the spatial dynamic change diagram of the predicted PM_2.5_ concentration can be established, from which the predicted PM_2.5_ concentration at any station in Shanghai can be obtained.

### 3.6 Result analysis and discussion

The PM_2.5_ concentration data of several observation stations in Shanghai are added to establish a spatial XGBoost model for prediction, through which the prediction level of PM_2.5_ concentration in each district is obtained. Finally, the kriging interpolation method is used to fill the adjacent areas of each observation station so as to obtain the prediction results of each observation station in Shanghai.

The findings are consistent with previous studies [2] that deep learning models fit well in integrating multiple factors. What's more, the stacking model could avoid overfitting if we adopt simple regression model [8], compared with using complex algorithm like cuckoo search. Variables from different fields could make us not take meteorological assumptions into consideration [4], which make the explanation of our model fitting with more situations. Some scholars have estimated hourly PM_2.5_ concentrations and found that the precision of the predicting results could be affected by the length of selected time span [3], while we utilize many air quality monitoring sites to construct spatial model to deal with defects caused by high frequent data. In some studies, some scholars give a large weight for those days in train set which have a high similarity in the meteorological conditions with the days in test set [5], but this method couldn't keep root mean square error at a constant level when adding more days into the test set. The reason lies in seasonal effect (usually they put days only in one season into the predicting set) and the model could be improved by using adaptive weighting method. What's more, more monitoring stations couldn't bring more precise result [9] and the mean square error could decrease if the stations include both urban area and suburban area. For the process of searching the best parameters of the XGBoost model, we take regularization term into consideration [11] and utilize particle swarm optimization algorithm to get global optimum, which largely improved the predicting result. To be specific, the model after parameter adjusting could perform better in some extremely pollutant days. This conforms to previous studies, as the high concentration PM_2.5_ value will be predicted more precisely [12] and, whether upward or downward, the trend of PM_2.5_ value could be described more accurately and smoothly. We utilize the inverse distance weighting (IDW) model to upgrade the precision of XGBoost model and the benefits of this method are as follow: on the one hand, we could add more information of air quality monitoring sites into our predicting model which could cover all the administrative regions in the city (Shanghai). On the other hand, it could also fill up the missing PM_2.5_ value and the result are consistent with those result in previous study [16]. There are also some other factors affecting the precision of predicting results. For example, air quality monitoring sites can be divided into national control points and self-built points [25] and this information could lead to utilizing different method to fill up the missing value of our data set. To the best of our knowledge, few studies have estimate seasonal PM_2.5_ concentration, potentially because they usually use data in only one year our put the period of only one quarter into the testing set, which may cause space heterogeneity of atmosphere contaminant in different season. For this reason [26], it's necessary to solve the error from contaminant distribution by constructing spatial model.

## 4 Results comparison and robustness analysis

### 4.1 Comparison of prediction effects before and after XGBoost model modification

#### 4.1.1 Prediction effect of XGBoost model before and after modification

After parameter adjustment, the optimal parameter settings of the XGBoost model can be determined as follows: min_child_weight is set to 3, n_estimate to 600, colsample_bytree to 0.9, max_dept to 3, gamma to 0.1, subsample to 0.8, and learning_rate to 0.1; the additional objective parameter selects the default term linear. The scores of the optimal values of these parameters are all over 80 %. The other models in the gradient boosting algorithm are selected as the comparison models of XGBoost. Taking the observation data set of Xujiahui as an example, the prediction effect of XGBoost model before and after modification is compared to demonstrate the superiority of the improved XGBoost model, as shown in Table 7.

**Table 7:** Prediction effect comparison between XGBoost models before and after modification, taking the predicted results of Xujiahui as an example.

Model	Group	MAE	Goodness of fit (*R*^2^)	RMSE	RPE
XGBoost	Trained	0.1801	90 %	3.2643	0.1587
	Test	0.1912	82.37 %	3.9	0.2065
Modified XGBoost	Trained	0.0785	98.05 %	2.5289	0.0821
	Test	0.1757	87.40 %	3.2976	0.1746

MAE: Mean absolute prediction error; RMSE: Root mean square error; RPE: Relative prediction error.

It can be seen that the modified XGBoost model has better performance in all evaluation dimensions.

#### 4.1.2 Comparison of XGBoost with other gradient boosting algorithms

Gradient boosting algorithms can be roughly divided into three types: LightGBM, XGBoost, and GBDT. Among them, XGBoost has the smallest average prediction error (0.1045), and LightGBM has the largest prediction error (0.1114). The difference between them is small. The goodness of fit is over 95 %, and the prediction effect is good. The prediction results of the three models are compared, as shown in Table 8.

**Table 8:** Error comparison among three models after introducing EMD decomposed sequences.

Model	MAE	Goodness of fit (*R*^2^)	RMSE	RPE
LightBGM	0.1114	98.86 %	3.62	6.53 %
GBDT	0.1669	98.53 %	4.1076	7.40 %
XGBoost	0.1045	99.25 %	2.9247	5.27 %

MAE: Mean absolute prediction error; RMSE: Root mean square error; RPE: Relative prediction error.

In general, XGBoost is superior in all evaluation dimensions, and the root mean square error of GBDT prediction is large. The performance of XGBoost on the test set is even better than that of the combined modified model, which indicates that the prediction ability of XGBoost has been greatly improved after parameter adjustment. Compared with GBDT, XGBoost improves the accuracy of the loss function. It replaces the first-order with the second-order Taylor expansion, and is superior to GBDT in the improvement of the regularization term. Moreover, the objective function is directly used as the loss function, which improves the flexibility of the model. In addition, XGBoost allows cross-validation in each iteration, and the later round can continue training on the training results of the previous round, which improves the operation efficiency. LightBGM is mainly to lower the value of loss function through the segmentation point algorithm, so as to achieve the purpose of improving training efficiency and reducing memory [38]. Thus, it is reasonable that the prediction accuracy of the XGBoost model after parameter adjustment is higher than that of LightBGM.

According to the comparison in Sections 4, 4.1.1.1.2, the modified XGBoost significantly outperforms the gradient boosting model before optimization and the XGBoost model without parameter adjustment.

### 4.2 Robustness analysis

In order to verify the robustness of the improved model, the data observed in Fengxian District are used again for model comparison with other conditions being unchanged. The results are listed in Table 9.

**Table 9:** Prediction effect comparison between XGBoost models before and after modification, taking the predicted results of Fengxian District as an example.

Model	Group	MAE	Goodness of fit (*R*^2^)	RMSE	RPE
XGBoost	Trained	0.2584	83.68 %	3.1887	0.2108
	Test	0.3381	73.63 %	4.6310	0.3021
Modified XGBoost	Trained	0.1164	98.15 %	2.3647	0.0851
	Test	0.3001	86.36 %	3.3305	0.2173

MAE: Mean absolute prediction error; RMSE: Root mean square error; RPE: Relative prediction error.

The results demonstrate that improved XGBoost model is robust and has better performance on two randomly selected datasets. Next, the number of observation stations is added to the model to investigate whether the prediction accuracy at a certain observation station can be improved. Table 10 is obtained by extending the distance matrix from 5 to 8 dimensions.

**Table 10:** Comparison of predicted results accuracy among extended distance matrices with different dimensions on test sets.

Matrix type	Area	MAE	Goodness of fit (*R*^2^)	RMSE	RPE
5 × 5	Xujiahui	0.1193	93.37 %	4.0797	0.1501
	Fengxian	0.3715	82.14	14.2832	0.5036
8 × 8	Xujiahui	1.7822	33.41 %	18.86	1.7977
	Fengxian	0.1923	78.74 %	7.2674	0.2562

MAE: Mean absolute prediction error; RMSE: Root mean square error; RPE: Relative prediction error.

The 5 most representative stations are selected by importance with XGBoost. When the matrix dimension increases to 8, the prediction effect for Xujiahui decreases (Fengxian rises slightly). With only 5 stations, the workload can be greatly reduced. Subsequently, the predictions of the spatial XGBoost model in urban and suburban areas are compared to see if there are differences.

Data in Table 11 shows that the prediction effect is not necessarily related to whether it is located in the urban area: the prediction result at Xujiahui is good, but that at Putuo is poor; the prediction result at Fengxian is good, while that at Qingpu is poor. Therefore, it is not necessary to consider the specific location of each observation station (urban or suburban) when using XGBoost to screen important observation stations.

**Table 11:** Comparison of test sets accuracy for between urban and suburb areas.

Observed area		MAE	Goodness of fit (*R*^2^)	RMSE	RPE
Urban	Xujiahui	0.1569	89.44 %	5.1481	0.1894
	Putuo	0.2916	61.47 %	10.3795	0.3697
Suburb	Fengxian	0.1019	93.99 %	3.8623	0.1362
	Qingpu	0.4081	51.58 %	11.1775	0.4465

MAE: Mean absolute prediction error; RMSE: Root mean square error; RPE: Relative prediction error.

## 5 Conclusions and limitations

This study innovatively adopted the adjusted XGBoost model associated with EEMD and Kriging interpolation to quantify and spatialized PM_2.5_ pollution. Based on the dataset, indicators representing air pollutant indicators, meteorological environmental indicators and macroeconomic indicators were analyzed for 16 case districts in Shanghai over a long-term period (2013–2021). Then, this study analyzes the seasonal effect and the PM_2.5_ distribution in Shanghai by using the model we constructed. The main findings can be summarized as follows.

1) Compared with the original model, the goodness of fit of the modified XGBoost model on the test set increased by 17 %, and the root mean square error decreased by 28 %, indicating that the performance of XGBoost model after parameter adjustment is significantly improved. Meanwhile, the improved model outperforms the other gradient boosting algorithms. In addition, the error of the constructed XGBoost fusion model at extreme value points is reduced. The reconstructed high-frequency sequence contains the most information of the original sequence, especially the fluctuation information, which means that the signal decomposition method can effectively obtain most information of original PM_2.5_ sequence when getting rid of useless information from some extreme points.
2) The variation of PM_2.5_ concentration in Shanghai has a significant seasonal (cyclical) effect, and its fluctuation period is 3 months (a quarter). The empirical results show that the PM_2.5_ level in Shanghai has shown a downward trend with fluctuations in recent years. The PM_2.5_ level in Shanghai is low in summer, but higher in winter. The air pollution shows obvious seasonal effect and presents a certain periodicity.
3) In terms of spatial distribution, the PM_2.5_ concentration in Putuo, Xujiahui, Yangpu and other urban areas is at a high level, and the pollution in Yangpu Industrial Zone is the most severe, and the PM_2.5_ concentration gradually decreases from center city to the surrounding areas.

This study has several limitations. For example, when the dimension of the distance matrix increases, the prediction accuracy of the model cannot be improved by either the method of inverse distance weighting or bandwidth selection. This indicates that before adding the data of each observation station, it is necessary to judge whether the data of this observation station is significantly different from those of other stations. Thus, further study can be carried out on these aspects.

## Data availability statement

Data will be made available on reasonable request. But some data haven't been deposited into the publicly available repository because they are not allowed to publicly accessed according to the request of the related website.

## CRediT authorship contribution statement

**Zidong Wang:** Data curation, Formal analysis, Investigation, Methodology, Resources, Software, Validation, Visualization, Writing - original draft, You Wu, Resources, Writing - review & editing. **Xianhua Wu:** Conceptualization, Funding acquisition, Supervision, Writing - review & editing.

## Declaration of competing interest

The authors declare that they have no known competing financial interests or personal relationships that could have appeared to influence the work reported in this paper.
